# Genome-Wide Dissection of the Heat Shock Transcription Factor Family Genes in *Arachis*

**DOI:** 10.3389/fpls.2017.00106

**Published:** 2017-02-06

**Authors:** Pengfei Wang, Hui Song, Changsheng Li, Pengcheng Li, Aiqin Li, Hongshan Guan, Lei Hou, Xingjun Wang

**Affiliations:** ^1^Biotechnology Research Center, Shandong Academy of Agricultural Sciences, Shandong Provincial Key Laboratory of Crop Genetic Improvement, Ecology and PhysiologyJinan, China; ^2^College of Life Sciences, Shandong Normal UniversityJinan, China

**Keywords:** heat shock transcription factor, peanut, abiotic stress, selective pressure, purifying selection

## Abstract

Heat shock transcription factors (Hsfs) are important transcription factors (TFs) in protecting plants from damages caused by various stresses. The released whole genome sequences of wild peanuts make it possible for genome-wide analysis of Hsfs in peanut. In this study, a total of 16 and 17 *Hsf* genes were identified from *Arachis duranensis* and *A. ipaensis*, respectively. We identified 16 orthologous Hsf gene pairs in both peanut species; however *HsfXs* was only identified from *A. ipaensis*. Orthologous pairs between two wild peanut species were highly syntenic. Based on phylogenetic relationship, peanut Hsfs were divided into groups A, B, and C. Selection pressure analysis showed that group B Hsf genes mainly underwent positive selection and group A Hsfs were affected by purifying selection. Small scale segmental and tandem duplication may play important roles in the evolution of these genes. Cis-elements, such as ABRE, DRE, and HSE, were found in the promoters of most *Arachis* Hsf genes. Five *AdHsfs* and two *AiHsfs* contained fungal elicitor responsive elements suggesting their involvement in response to fungi infection. These genes were differentially expressed in cultivated peanut under abiotic stress and *Aspergillus flavus* infection. *AhHsf2* and *AhHsf14* were significantly up-regulated after inoculation with *A. flavus* suggesting their possible role in fungal resistance.

## Introduction

Abiotic stresses, including heat, cold, drought, and salinity, affect plant growth and development and cause serious loss of crop production (Wang et al., 2004; Al-Whaibi, 2011; Qiao et al., 2015). As sessile organisms, plants could not change their locations when facing such stress conditions (Guo et al., 2016). However, plants have evolved adaptation strategies to these stresses (Scharf et al., 2012; Guo et al., 2016). Transcription factors play a crucial role in stress tolerance by regulating the expression of thousands of genes under unfavorable conditions (Schwechheimer and Bevan, 1998; Kreps et al., 2002; Shinozaki and Yamaguchi-Shinozaki, 2007; Wang et al., 2014). Plant heat shock transcription factors (Hsfs) are important transcription factors (TFs) in protecting plants from heat stress and other stresses, including cold, salinity, and drought (Kotak et al., 2007a; Swindell et al., 2007; Hu et al., 2015). Hsfs were found in eukaryotes from yeast to humans (Ritossa, 1962; Tanabe et al., 1997; Akerfelt et al., 2010). Hsfs could protect cells from extreme proteotoxic damage via the activation of related genes (Dalton et al., 2000; Akerfelt et al., 2010; Yang et al., 2014; Jaeger et al., 2016). Studies showed that Hsfs are also involved in plant growth and development (Almoguera et al., 2002; Díaz-Martín et al., 2005; Kotak et al., 2007b).

Hsfs regulate heat shock response via activating the expression of heat shock protein (HSP) genes by binding to the heat shock elements (HSEs) (Pelham, 1982; Akerfelt et al., 2010). The sequences and geometrys of HSEs (5′-AGAAnnTTCT-3′) are variable (Guertin et al., 2012; Mendillo et al., 2012; Vihervaara et al., 2013). Hsfs could also bind to SatIII repeat element, 5′-cgGAAtgGAAtg-3′ (Grady et al., 1992). Like many other transcription factors, Hsfs have an N-terminal DNA binding domain (DBD) and followed by an oligomerization domain (OD). OD is composed of two hydrophobic heptad repeats (HR-A/B) which allows homo- and hetero-multimerization (Peteranderl et al., 1999; Nover et al., 2001; Baniwal et al., 2004). Certain Hsfs contained nuclear location signal (NLS) domain, nuclear export signal (NES), and C-terminal activation (AHA) domain (Döring et al., 2000; Hsu et al., 2003; Maere et al., 2005). Based on the structural characteristics of HR-A/B domain and the phylogenetic relationship, plant Hsfs are divided into A, B, and C groups (Von Koskull-Döring et al., 2007; Wang et al., 2014; Yang et al., 2014). Additional sequences were found in HR-A/B domain of group A and C, but not in group B Hsfs (Nover et al., 2001; Schmidt et al., 2012; Wang et al., 2014; Yang et al., 2014).

Only the active Hsfs are capable of recognizing and binding to the promoters of target genes. The inactive monomer could be converted into active oligomer under variety of stress conditions (Hartl and Hayer-Hartl, 2002; Wang et al., 2012; Li et al., 2014). There are only a few Hsf genes in yeast and animals, while 20–50 Hsf genes were found in plants (Scharf et al., 2012; Lin et al., 2014; Qiao et al., 2015). Hsf genes were identified in many plants and expressed in various tissues at different developmental stages during different stress conditions (Giorno et al., 2012; Chung et al., 2013; Xue et al., 2014).

Peanut (*Arachis hypogaea* L.) is an important oil crop in the world. In developing countries, peanuts were rain-fed, so it is important to study the drought stress tolerance of peanut (Ramu et al., 2015). *Aspergillus flavus* produces potent mycotoxins known as aflatoxins that could cause serious health concerns (Zhang et al., 2015). It is unknown on the role of Hsf genes in peanut response to abiotic stresses and *A. flavus* infection.

Cultivated peanut is an allotetraploid (AABB, 4*n* = 4*x* = 40) originated from a single hybridization and genome duplication event between two wild type diploid peanuts (AA and BB genomes) (Kochert et al., 1996; Freitas et al., 2007; Moretzsohn et al., 2013; Wang et al., 2016). Recently, the whole genome sequencing of the two ancestral species (*A. duranensis* and *A. ipaensis*) have been completed (Bertioli et al., 2016; http://peanutbase.org/). Here, we genome-widely identified and analyzed the *Hsf* genes from two wild peanuts species: *A. duranensis* (AA genome) and *A. ipaensis* (BB genome), respectively. We analyzed the gene duplication events in the wild peanut species, the difference of selection pressure in A, B, and C group of *Arachis* Hsfs, and the structures of these proteins. Our results provide basic information for further understanding the functional divergence and evolution of *Arachis* Hsfs. We also applied the knowledge gained from wild species to cultivated one to understand their possible functions on peanut response to abiotic and biotic stress.

## Materials and methods

### Data collection and identification of *Hsf* genes

The genome sequence data of two wild peanut species (AA and BB genomes) were obtained from the peanut genome database (http://peanutbase.org/). The conserved domains of Hsfs are Hsf-type DBD domain. The HMM ID of this domain is PF00447 in the pfam database (http://pfam.xfam.org/). The amino acid sequences of HMMs were used as queries to identify all possible Hsf protein sequences in AA and BB genome database using BLASTP (*E* < 0.001). SMART software (http://smart.embl-heidelberg.de/) was used to identify integrated DBD domain and (HR-A/B) domain in the putative peanut Hsfs. Candidate proteins without integrated DBD domain and HR-A/B domain were removed. NLS domains in peanut Hsfs were predicted using cNLS Mapper software (http://nls-mapper.iab.keio.ac.jp/cgi-bin/NLS_Mapper_form.cgi). NES domains were predicted using NetNES 1.1 server software (http://www.cbs.dtu.dk/services/NetNES/). AHA domains were predicted based on the conserved-type AHA motif sequence FWxxF/L, F/I/L (Kotak et al., 2004). Protein isoelectric point (pI) and molecular weight (Mw) were analyzed using Expasy software (http://web.expasy.org/compute_pi/).

The genome, protein, and cDNA sequences were collected from the related genome databases for the following additional plant species: *Arabidopsis thaliana* (http://www.plantgdb.org/AtGDB/), *Glycine max* (http://www.plantgdb.org/GmGDB/), *Lotus japonicus* (http://www.plantgdb.org/LjGDB/), *Medicago truncatula* (http://www.plantgdb.org/MtGDB/), *Cajanus cajan* (http://gigadb.org/dataset/100028) and *Cicer arietinum* (http://nipgr.res.in/CGAP/home.php).

### Orthologous gene identification and structure analysis

Orthologous gene pairs were identified according to (1) the best-hit between *A.duranensis* and *A. ipaensis*, (2) the position in the phylogenetic tree (bootstrap value >50), and (3) identity between ortholougs gene pairs (>90%). Circos software was used to plot the chromosomal location (Krzywinski et al., 2009). Gene Structure Display Server 2.0 (http://gsds.cbi.pku.edu.cn/) was used to plot the gene structure.

### Analysis of synteny

Intraspecies synteny analysis of AA or BB genome and interspecies synteny analysis between AA and BB genomes were based on comparison of 100 kb blocks of chromosome containing Hsf genes according to previous reports (Sato et al., 2008; Zhang et al., 2011; Lin et al., 2014). Hsf genes were set as anchor points according to their chromosome locations. Blocks were identified by local all-vs-all BLASTN (*E* < 10^−20^). In intraspecies analysis, when four or more homology genes were detected, these two blocks were considered to be originated from a large-scale duplication event (Zhang et al., 2011; Lin et al., 2014). In interspecies analysis, when three or more conserved homology genes were detected, these two blocks were considered syntenic blocks (Sato et al., 2008; Lin et al., 2014; Wang et al., 2016).

### Multiple sequence alignment and phylogenetic analysis

Protein multiple sequence alignment was performed using online software Clustal Omega (http://www.ebi.ac.uk/Tools/msa/clustalo). Neighbor-Joining (NJ) trees were constructed using MEGA 6.0 with protein sequences. To support the calculated relationship, 1000 bootstrap samples were generated. A total of 21 *A. thaliana* Hsfs (Scharf et al., 2012), 11 *M. truncatula* Hsfs, 10 *L. japonicus* Hsfs, 16 *C. cajan* Hsfs (Lin et al., 2014), 11 *C. arietinum* Hsfs, and 40 *G. max* Hsfs were included in the phylogenetic analysis (Poisson correction, pairwise deletion, and bootstrap = 1000 replicates; Xue et al., 2014). All Hsfs used in this study was listed in the Table S1.

### Gene duplication analysis

Two standards for duplication gene identification were used. High-stringency standard: coding protein pair with ≥50% identity and covering ≥90% protein length. Low-stringency standard: protein pair with ≥30% identity and covering ≥70% protein length (Rizzon et al., 2006). Tandem duplication of genes was marked according to the previously described method (Yuan et al., 2015). Chromosome segmental or large scale duplication of genes was identified based on the intraspecies synteny (Zhang et al., 2011; Lin et al., 2014; Qiao et al., 2015).

### Protein structure analysis and homology modeling

SWISS-MODLE (http://www.swissmodel.expasy.org/interactive) was used to calculate secondary structure and build three-dimensional structure of proteins. The templates for building protein 3D model were selected in PDB database based on the best identity. Protein 3D models were selected based on the best global model quality estimation (GMQE). Homology modeling templates included 5d5v.1 (monomer of DBD domain), 5d5v.1 (homo-dimer of DBD domain interacted with SalIII), 5d5u.1 (homo-dimer DBD domain interacted with HSE), 4r0r.1.A (monomer of HR-A/B domain) and 4r0r.1 (homo-trimer of HR-A/B).

### Analysis of selective pressure

Codeml program under PAML (phylogenetic analysis maximum likelihood) version 4.7 software (Yang, 2007) was used to detect whether the Hsf genes underwent positive selection. In PAML, six site models, M0 (one ratio), M1a (neutral), M2a (positive selection), M3 (discrete), M7 (beta) and M8 (beta and ω) could be applied to selection pressure analysis. Positive selection sites could be identified by the comparison of M0-M3, M1a-M2a, and M7-M8 (Yang et al., 2000).

### Analysis of cis-acting regulatory elements in promoter

Plantcare software (http://bioinformatics.psb.ugent.be/webtools/plantcare/html/) was used to predict cis-acting regulatory elements.

### Plant materials, stress treatments, and RNA isolation

Cultivated peanut cv. Luhua-14 was used in this study. Eleven-day-old peanut seedlings were subjected to drought (removed from wet medium and kept in air on filter paper), cold (4°C) and high temperature (42°C) treatment. Leaf samples were collected at 0, 1, and 6 h after treatment and immediately frozen in liquid nitrogen. Leaf samples without treatment were used as control. Peanut seeds inoculated with *A. flavus* for 3 days were collected and seeds without *A. flavus* inoculation were used as control according to a previous report (Zhang et al., 2015). RNAs were isolated by CTAB method according to a previous method (Wang et al., 2016). For reverse transcription, the first-strand cDNA was synthesized with an oligo (dT) primer using a PrimeScript™ first-strand cDNA synthesis kit (TaKaRa). Three technical replicates were carried out in this study.

### Gene expression analysis

Quantitative real time PCR (qRT-PCR) was performed using the FastStart Universal SYBR Green Master (ROX) with ABI™ 7500. The qRT-PCR program was set as the following: 95°C for 30 s, followed by 40 cycles of 95°C for 5 s, 60°C for 30 s. Relative gene expression levels were calculated using the ^ΔΔ^CT method. The primers for qRT-PCR were provided in the Table S2. *T*-test was used to analyze the significance.

## Results

### Identification of Hsf genes in wild peanut species

**Figure 1:**
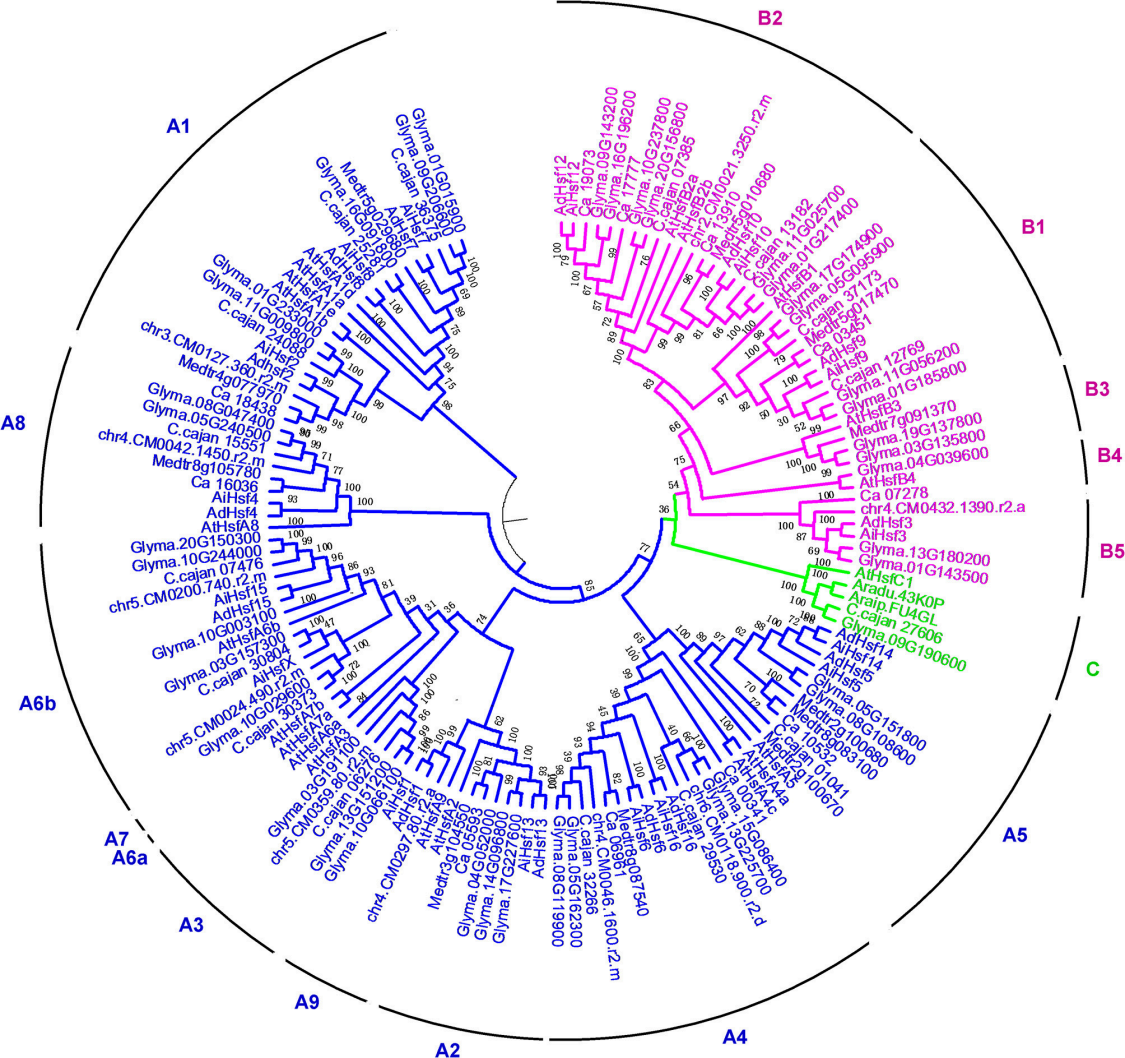
**Neighbor-joining phylogenic analysis of Hsfs**.

The amino acid sequences of Hsfs were extracted from AA and BB wild peanut genome database using the BLASTP program. The amino acid sequences of Hsf DBD domains (Pfam: PF00447) were used as queries. From AA and BB genomes, we identified 16 and 17 Hsf genes, respectively. The polypeptide lengths of Hsfs varied from 209 to 656 aa in *A. duranensis* and from 282 to 514 aa in *A. ipaensis. A. thaliana* Hsf family were often employed as reference to classify Hsf family in other plant species (Scharf et al., 2012; Li et al., 2014; Wang et al., 2014; Qiao et al., 2015). We employed Hsfs from *A. thaliana* and other species to construct phylogenetic tree together with Hsfs in two wild peanut species. In this study, 21 *A. thaliana* Hsfs, 11 *M. truncatula* Hsfs, 10 *L. japonicus* Hsfs, 16 *C. cajan* Hsfs, 11 *C. arietinum* Hsfs, and 40 *G. max* Hsfs were used for phylogenetic tree construction (Figure 1). These Hsfs were divided into A, B, and C groups that was consistent with previous studies (Scharf et al., 2012; Li et al., 2014; Lin et al., 2014; Wang et al., 2014; Qiao et al., 2015). Group A was divided into 10 clusters, group B was divided into five clusters, and group C contained only one cluster. Clusters in the group A were named as A1–A5, A6a, A6b, A7–A9. Clusters in the group B were named as B1–B5. B5 cluster was not presented in *Arabidopsis*; however, B5 cluster was identified in many leguminous species including wild peanut species. In wild peanut species, A3, A6a, A7, B3, and B4 clusters were absent (Figure 1). Orthologous of all 16 AA genome Hsfs were found in the BB genome with >90% identity (Table S3).

**Figure 2:**
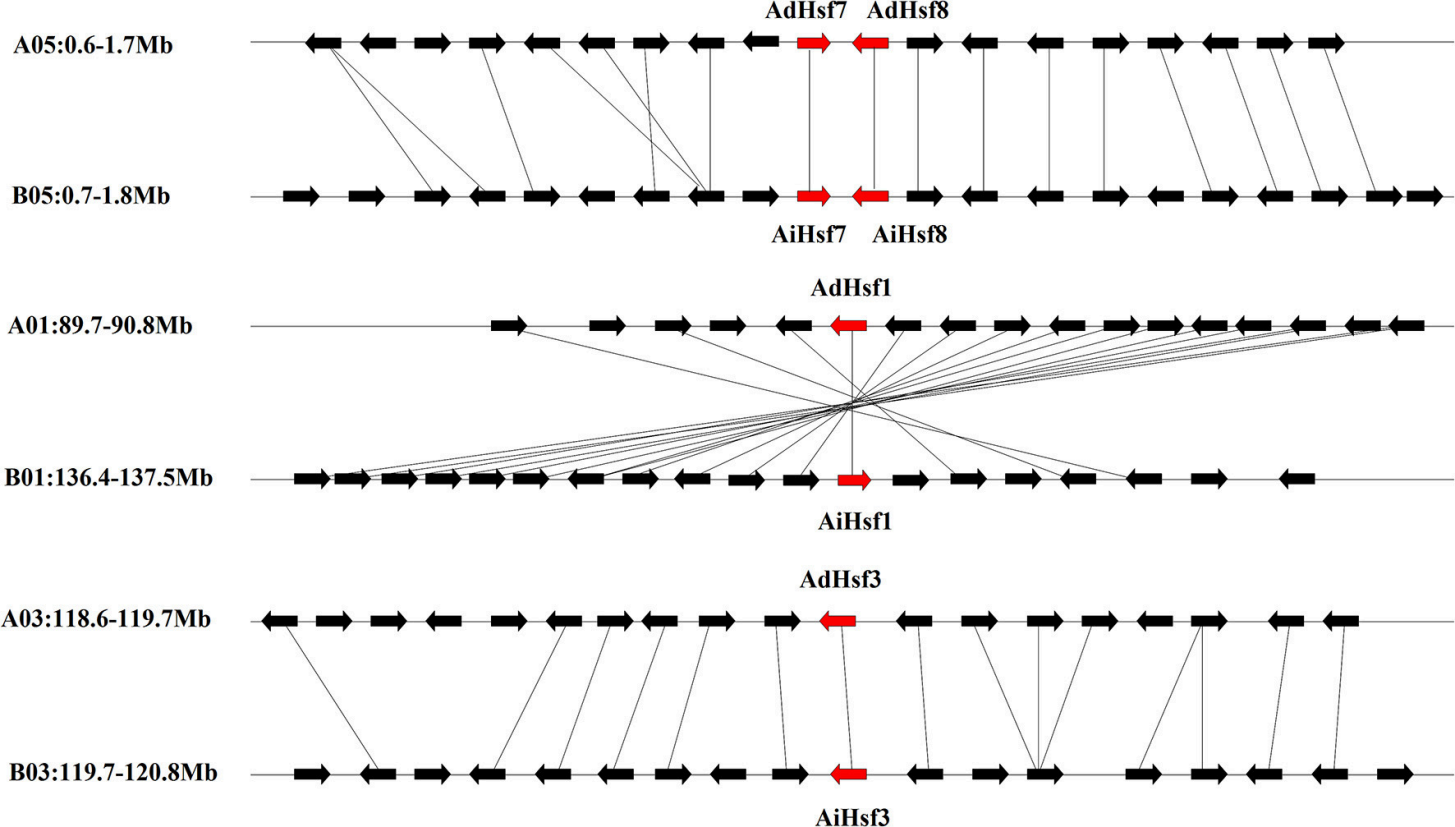
**Syntenic chromosomal segments between genes flanking AA genome ***Hsfs*** and their orthologous genes in BB genome**. Red arrows represent *Hsf*, black arrows represent flanking genes.

**Figure 3:**
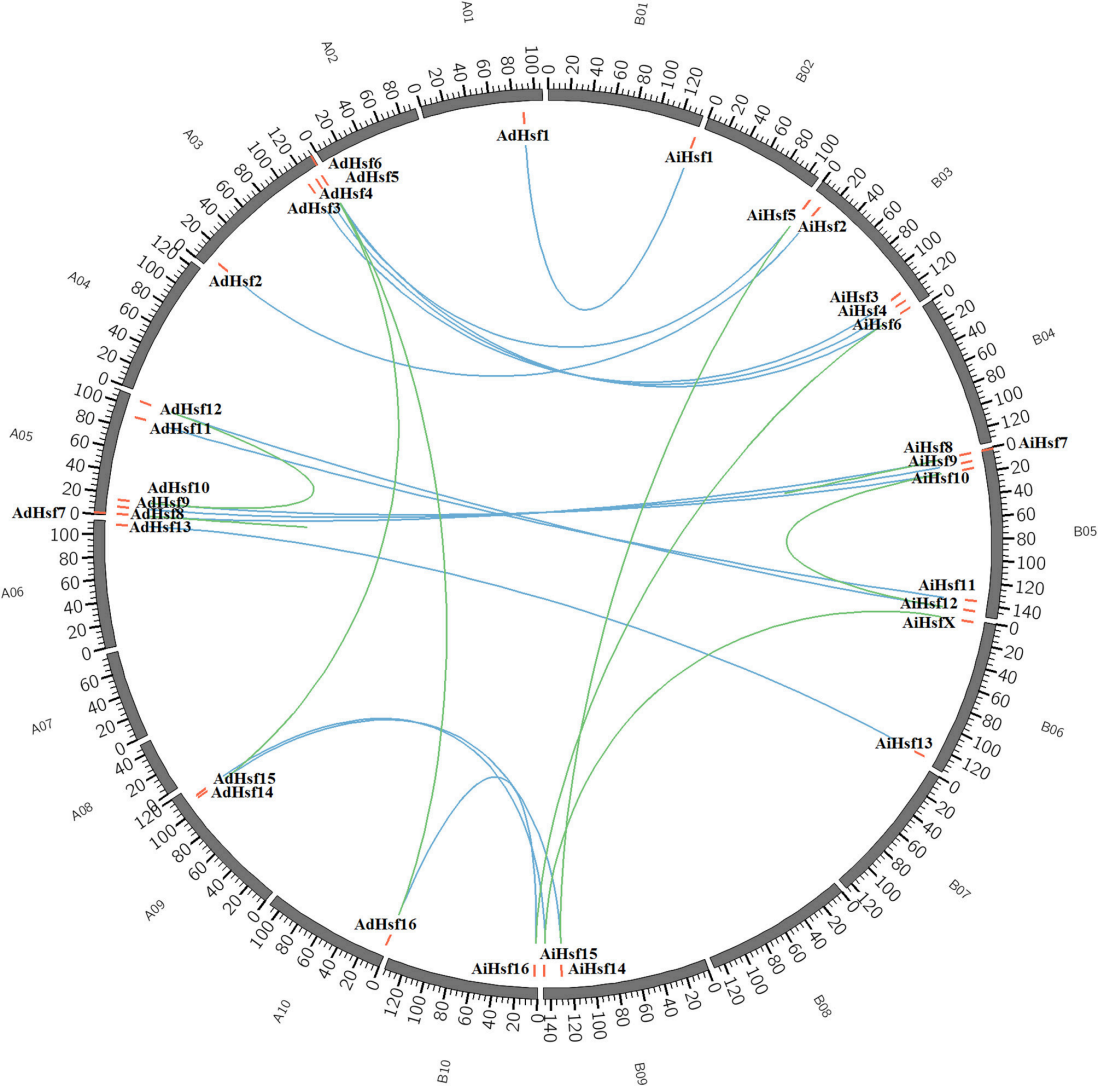
**Genome location of ***Hsf***s in AA and BB genome peanuts**. AA and BB wild peanut *Hsfs* were marked with red short lines on their genome location. Duplicated gene-pairs linked with green long curves and orthologous gene-pairs linked with blue curves.

Interspecies synteny analysis showed that high level synteny was maintained between AA and BB genomes (Figure 2). This synteny analysis supported the identification of orthologous-pairs of Hsfs between AA and BB genomes. The nomenclature of AA genome Hsfs was based on their chromosome location order, AdHsf1-16. BB genome Hsfs were named based on their orthologous genes in AA genome AiHsf1-16 and AiHsfX. The orthologous gene of AiHsfX (Araip. A5C77) was not found in AA genome. The gene IDs and physical locations information of wild peanut Hsf genes were showed in Table 1, Figure 3.

**Table 1:** *****Hsf***s identified in wild type peanuts**.

**Gene name**	**Chromosome location**	**Gene ID**
*AdHsf12*	Aradu.A05:106,432,571.106,434,237	*Aradu.H5REB*
*AdHsf9*	Aradu.A05:7,998,864.8,000,946	*Aradu.S4DGV*
*AdHsf10*	Aradu.A05:14,375,242.14,377,500	*Aradu.NTQ7W*
*AdHsf1*	Aradu.A01:90,085,291.90,088,131	*Aradu.5S8J3*
*AdHsf2*	Aradu.A03:8,559,905.8,562,946	*Aradu.N3N49*
*AdHsf16*	Aradu.A10:1,011,979.1,014,302	*Aradu.H8ZFN*
*AdHsf15*	Aradu.A09:110,970,626.110,972,271	*Aradu.MA5WH*
*AdHsf6*	Aradu.A03:134,564,420.134,565,759	*Aradu.RVF1V*
*AdHsf7*	Aradu.A05:1,337,984.1,342,330	*Aradu.G1NHG*
*AdHsf4*	Aradu.A03:127,573,853.127,576,100	*Aradu.N1DAK*
*AdHsf5*	Aradu.A03:133,622,480.133,625,670	*Aradu.R0U9B*
*AdHsf11*	Aradu.A05:91,450,473.91,451,748	*Aradu.43K0P*
*AdHsf14*	Aradu.A09:108,105,828.108,110,202	*Aradu.RFN6Q*
*AdHsf8*	Aradu.A05:1,343,331.1,346,309	*Aradu.95F2Q*
*AdHsf3*	Aradu.A03:119,139,289.119,141,972	*Aradu.X3DNX*
*AdHsf13*	Aradu.A06:108,820,015.108,822,104	*Aradu.RY508*
*AiHsf7*	Araip.B05:1,259,505.1,263,848	*Araip.T98BR*
*AiHsf12*	Araip.B05:146,768,456.146,770,030	*Araip.8V58J*
*AiHsf9*	Araip.B05:8,412,092.8,414,222	*Araip.90YM8*
*AiHsf10*	Araip.B05:15,070,998.15,073,228	*Araip.K80UC*
*AiHsf1*	Araip.B01:137,050,298.137,053,902	*Araip.DCD5Q*
*AiHsf2*	Araip.B03:11,777,014.11,780,056	*Araip.U7I8R*
*AiHsf16*	Araip.B10:2,967,059.2,969,380	*Araip.4A18K*
*AiHsf15*	Araip.B09:145,996,176.145,998,569	*Araip.24AK5*
*AiHsfX*	Araip.B06:1,771,852.1,780,911	*Araip.A5C77*
*AiHsf6*	Araip.B03:135,626,726.135,628,064	*Araip.2D8LN*
*AiHsf4*	Araip.B03:128,239,020.128,240,481	*Araip.3P0PJ*
*AiHsf5*	Araip.B03:889,869.893,296	*Araip.Z1XB6*
*AiHsf11*	Araip.B05:137,352,977.137,354,281	*Araip.FU4GL*
*AiHsf3*	Araip.B03:120,142,696.120,145,308	*Araip.UGV6F*
*AiHsf8*	Araip.B05:1,265,312.1,268,368	*Araip.SMI4I*
*AiHsf14*	Araip.B09:130,155,536.130,159,602	*Araip.G2FC5*
*AiHsf13*	Araip.B06:133,072,149.133,074,221	*Araip.B0RQS*

*These gene ID could be searched on web (http://peanutbase.org/keyword_search)*.

### Duplication of Hsf genes in peanut

Duplicated gene-pairs were found in both AA and BB genomes, including high-stringency standard duplicated gene-pairs *AdHsf5-AdHsf14, AdHsf6-AdHsf16* in AA genome and *AiHsf5-AiHsf14, AiHsf6-AiHsf16, AiHsf7-AiHsf8* in BB genome, low-stringency standard duplicated gene-pairs *AdHsf7-AdHsf8* in AA genome and *AiHsf15-AiHsfX* in BB genome. Intraspecies synteny analysis showed that the duplicated gene-pair blocks were not collinear. No chromosome segmental or large scale duplication gene pairs were identified. *AiHsf7-AiHsf8* and *AdHsf7-AdHsf8* were identified as tandem duplicated gene-pairs.

### Features of *Hsfs* in wild peanut species

Most members of Hsf gene families in both AA and BB genomes contained one intron and two exons. However, *AdHsf7* contained three exons and *AdHsf14* contained four exons in the AA genome, *AiHsf15* contained three exons, *AiHsf14*, and *AiHsfX* contained four exons in the BB genome. *AdHsf14* contained four exons, while its duplicated gene *AdHsf5* contained only two exons. Intronless Hsfs were also found in both AA and BB genomes (Figure S1).

**Figure 4:**
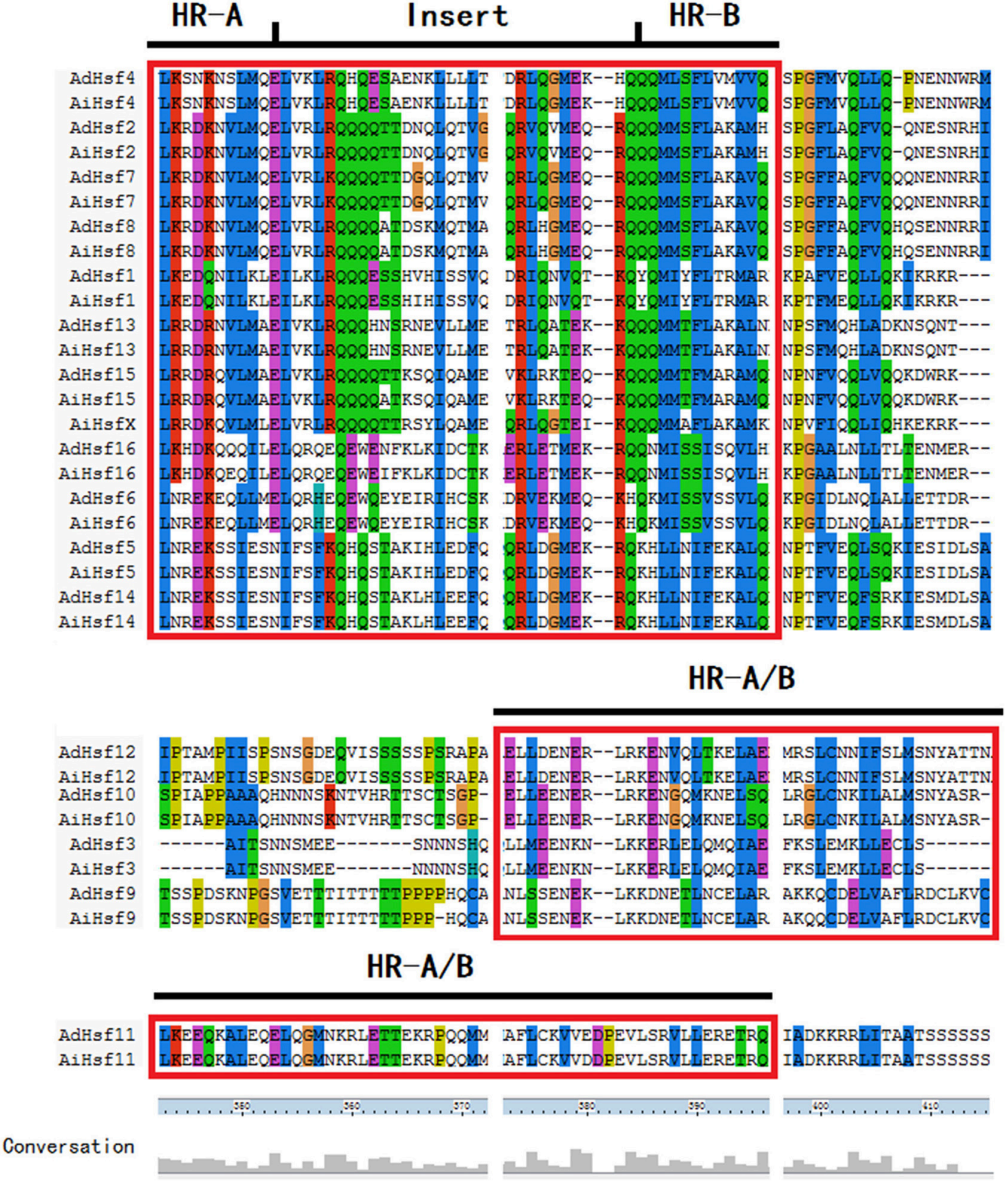
**HR-A/B domain in peanut Hsfs**.

HR-A/B domain is critical for one Hsf interacting with other Hsfs to form trimer through a helical coiled-coil structure (Scharf et al., 2012; Jaeger et al., 2016; Neudegger et al., 2016). Similar to other plant Hsfs, group A Hsfs have an insertion between HR-A and HR-B regions in peanut. However, this insertion was not found in the group B Hsfs. In *Arachis*, the sequence of group B Hsf HR-A/B was not conserved compare with that in group A (Figure 4). The DBD domains were conserved in two wild peanut species. The most conserved motif of DBD domains were “FSSFI/VRQLNT/I” in peanut (Figure S2).

### The 3D structure of *Hsfs* in wild peanut species

**Figure 5:**
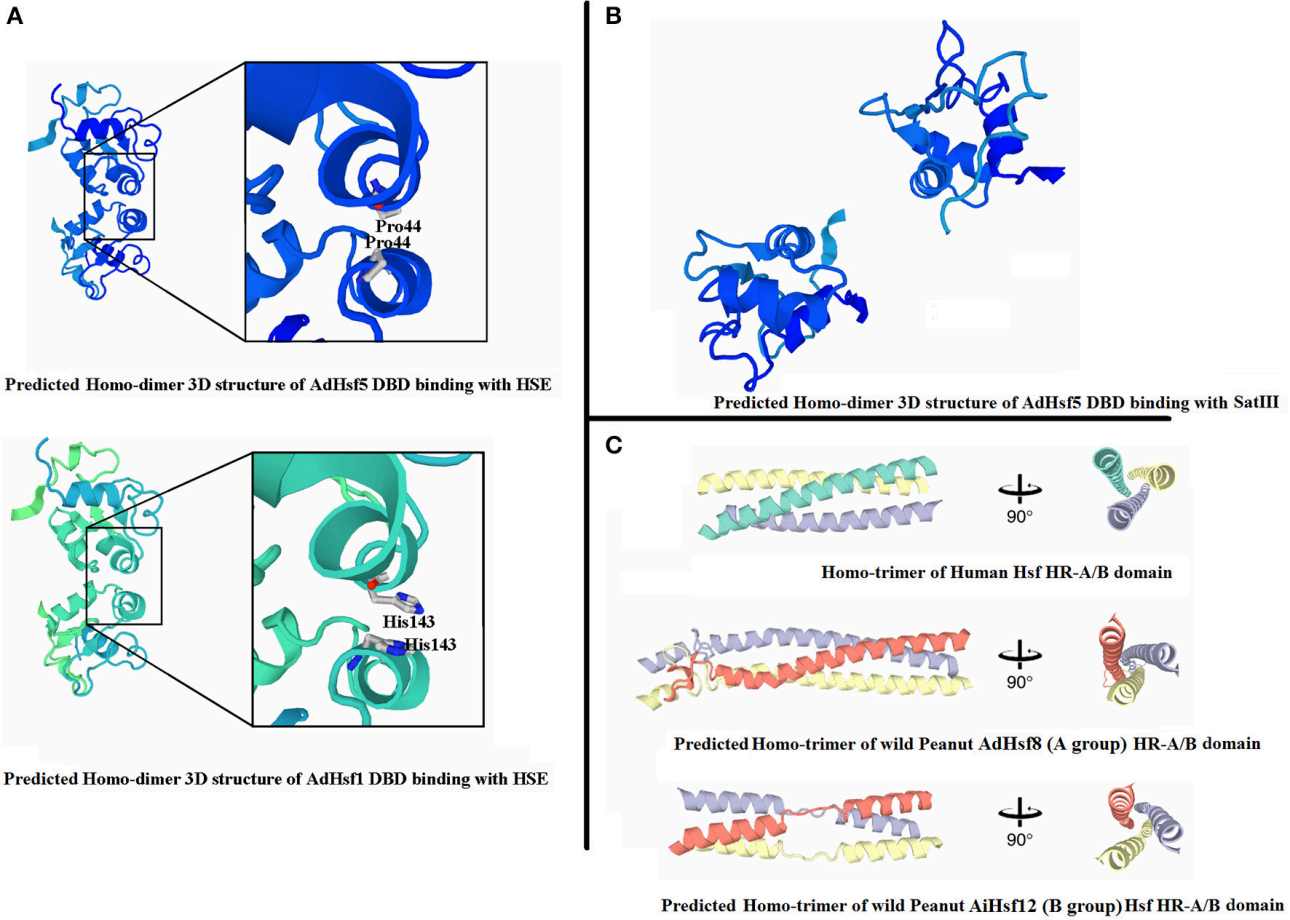
**3D structure of peanut Hsfs. (A)** Represents 3D structures of peanut Hsf DBD domains binding with HSE. **(B)** Represents 3D structures of peanut Hsf DBD domains binding with SatIII. **(C)** Represents homo-trimer of Hsf HR-A/B domain.

The predicted 3D structures of BDB domain of all AA and BB Hsfs were similar to that of human Hsf BDB (Figure 5A). The predicted 3D structures of HR-A/B domain of AA and BB Hsfs were also similar to the human Hsfs (Figure 5C). The 3D structures of BDB domain of peanut orthologous were highly conserved.

When adjacent DBD molecules bound to HSE element, two DBD molecules formed symmetrical protein-protein interaction involving the helix α2. The closest intermolecular contact occurred between the Gly50 residues located at the N-terminal end of the α2 helices in chordate Hsfs. Gly50 is conserved and is surrounded by Gln49 and Gln51 in chordate Hsfs (Neudegger et al., 2016). In peanut, we predicted that the closest intermolecular contact residues by homologous comparison and 3D model comparison. The results showed that the closest contact residues were not conserved between chordate Hsfs and peanut Hsfs. For example, in AdHsf1 and AdHsf5, the predicted closest intermolecular contact occurred between the residues His143 (Figure 5A). We also built models that DBD domain of AA and BB wild peanut Hsfs bound to SatIII element. The result showed that the predicted dimer structures of DBD-DBD interaction for binding to SatIII element and HSE element were distinct (Figure 5B).

### Selective pressure analysis of *Hsfs* in wild peanut species

Site models were used to detect whether different groups of *Hsfs* were under different selective pressure in peanuts. Group C *Hsfs* contained only one gene, it could not be analyzed. M0 showed that both *AdHsfs* and *AiHsfs* in group A underwent strong purifying selection (ω = 0.31723 in AA genome and ω = 0.40488 in BB genome; Table S4). Interestingly, in group B, both *AdHsfs* and *AiHsfs* were underwent positive selection (ω = 1.69713 in AA genome and ω = 1.95226 in BB genome). M0 vs. M3, M1a vs. M2a and M7 vs. M8 comparisons detected 399 positive selection sites in group B *AdHsfs* (*P* < 0.05) and 382 positive selection sites in group B *AiHsfs* (*P* < 0.001; Table S4). The identification of these positive selection sites in group B *Hsfs* indicated extensive functional diversity and structural variation (Wang et al., 2016).

### Cis-acting regulatory element analysis of peanut *Hsf* promoter

*In silico* survey of the putative cis-acting regulatory elements in the 1500 kb promoter region of *Hsfs* was performed. The majority of *Hsf* promoters contained HSE elements. HSE was not found in *AdHsf3, AdHsf12, AdHsf14, AiHsf1*, or *AdHsf11* promoters. Many *Hsf* promoters except *AiHsf2* contained abiotic stress responsive element such as MBS (drought inducible), LTR (low temperature responsive), and ARE elements (anaerobic induction). RNA-seq data showed that two *A. duranensis* Hsf genes (Aradu.X3DNX, AdHsf3, and Aradu.5S8J3, AdHsf1) were up-regulated significantly under drought stress (log_2_ *FC* > 2, *FDR* < 0.05) (Guimarães et al., 2012; Brasileiro et al., 2015). Phytohormone-induced elements, such as ERE element (ethylene-responsive element), AuxRR-core or TGA-element (auxin responsive), GARE-motif, or P-box element (gibberellin-responsive), ABRE element (ABA responsive), TCA-element (salicylic acid responsive), and TGACG-motif or CGTCA-motif element (MeJA-responsive) were found in some Hsf promoters. Five *AdHsfs* (*AdHsf2, AdHsf4, AdHsf16, AdHsf8*, and *AdHsf6*) and two *AiHsfs* (*AdHsf11* and *AdHsf10*) contained fungal elicitor responsive elements. Promoters of orthologous genes between AA and BB genomes were similar (Table S5).

### Expression of *Hsfs* in various tissues in cultivated peanut

**Figure 6:**
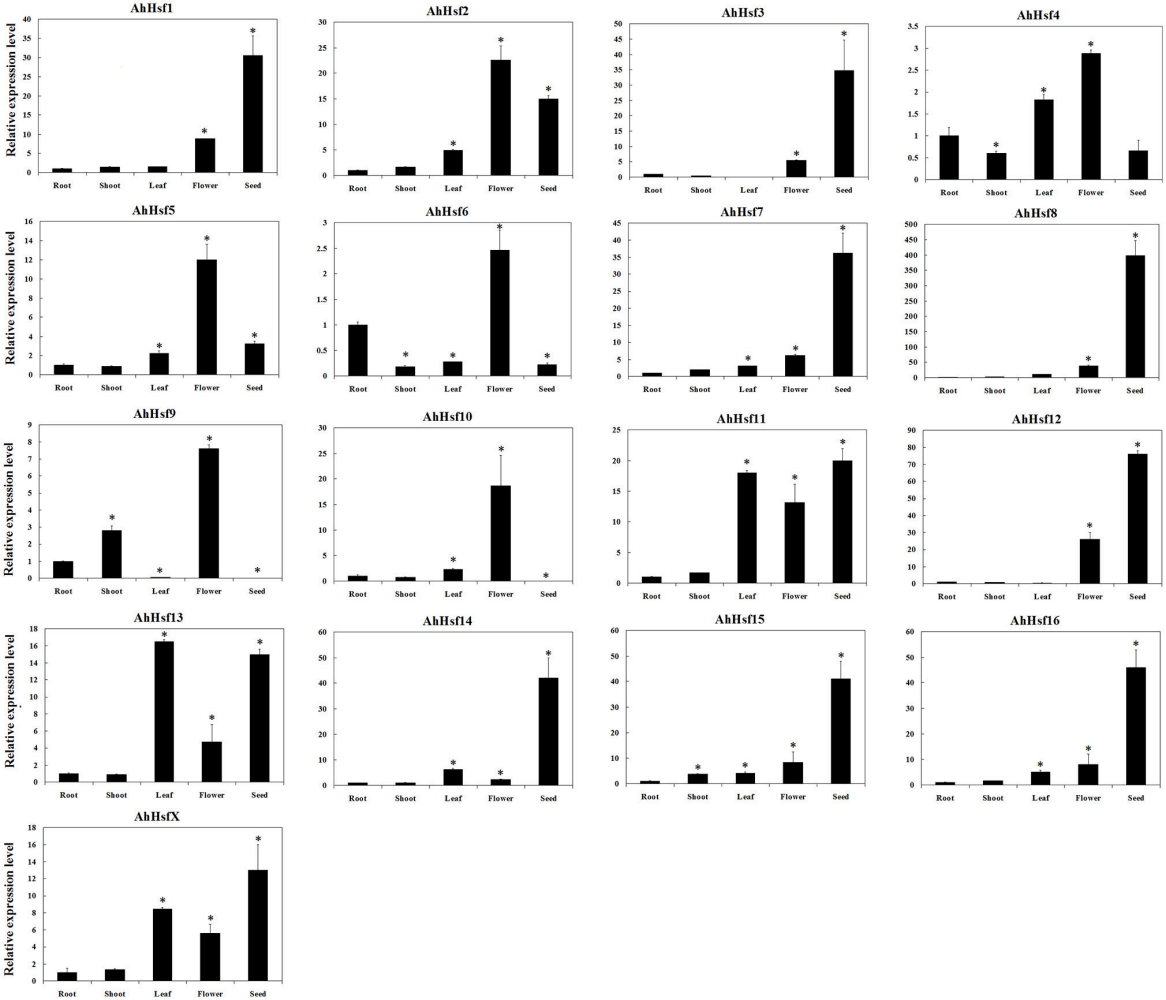
**Relative expression levels of ***Hsfs*** in different tissues in cultivated peanut**. *T*-test was used to perform analysis of significance. ^*^ represents significantly difference (*P* < 0.05) compared with control (0 h).

We used *Hsfs* of wild peanut species as queries to identify *Hsfs* in cultivated peanut species from transcriptome and genomic sequences (unpublished data). Totally, 17 *Hsfs* were identified in cultivated peanut species and named as *AhHsf1- AhHsf16 and AhHsfX*. The sequences of these genes were similar to their orthologous genes in wild peanut species (Table S6). To predict the possible function of these genes in cultivated peanut, the expression of these genes was investigated by qRT-PCR. Results showed that *AhHsf1, 3, 7, 8, 11, 12, 14, 15, 16*, and *X* were expressed predominantly in seeds, while the expression of *AhHsf9* and *10* was not detected in seeds. *AhHsf2, 4, 5, 6, 9*, and *10* were highly expressed in flower. The expression of *AhHsf1, 7, 12, 15*, and *16* was higher in flower than that in root, shoot or leaf. The expression of *AhHsf11, 13*, and *X* was higher in leaf than that in root, shoot or flower. The expression of *AhHsf4* and *AhHsf6* was higher in root than that in shoot or seed. The expression level of *AhHsf9* was higher in shoot than that in leaf or seed (Figure 6).

### *Hsf* expression in response to various stresses in cultivated peanut

**Figure 7:**
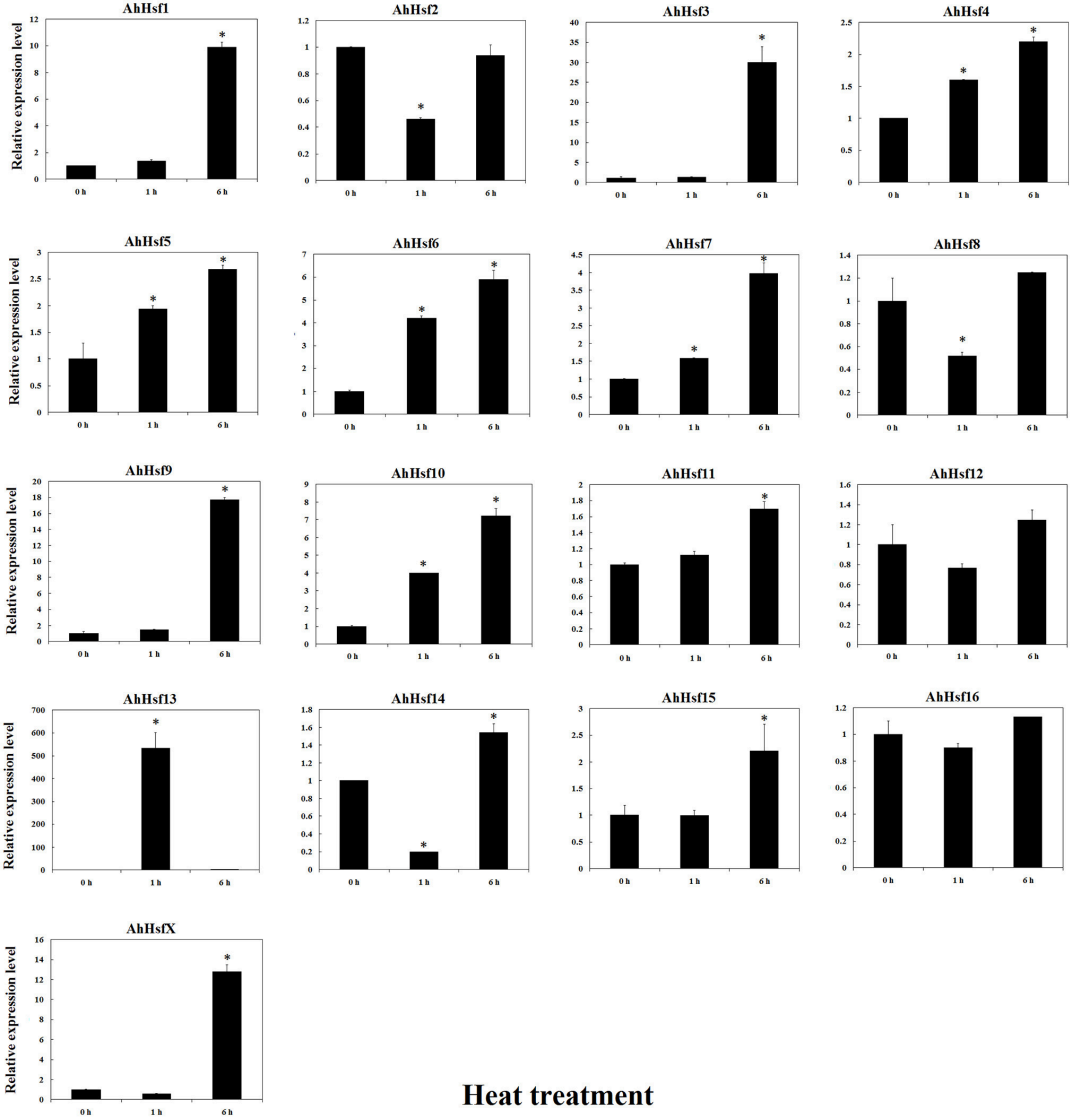
**Relative expression levels of ***Hsfs*** under heat stress in cultivated peanut**. *T*-test was used to perform analysis of significance. ^*^ represents significantly difference (*P* < 0.05) compared with control (0 h).

The expression of *AhHsf* was analyzed under high temperature, drought and low temperature by qRT-PCR. The expression levels of most *Hsfs* (*AhHsf1, 3, 4, 5, 6, 7, 9, 10, 11, 13, 14, 15*, and *X*) were up-regulated under high temperature. The expression of *AhHsf1, 3, 9, 15*, and *X* was up-regulated up to ~9-folds after 6 h treatment with 42°C. *AhHsf4, 5, 6, 10, 11*, and *13* could response rapidly to high temperature, and up-regulated after 1 h treatment. The expression of *AhHsf4, 5, 6, 10*, and *11* was continuously increased during 1–6 h of 42°C treatment. The expression of *AhHsf13* was decreased at 6 h after 42°C treatment (Figure 7).

**Figure 8:**
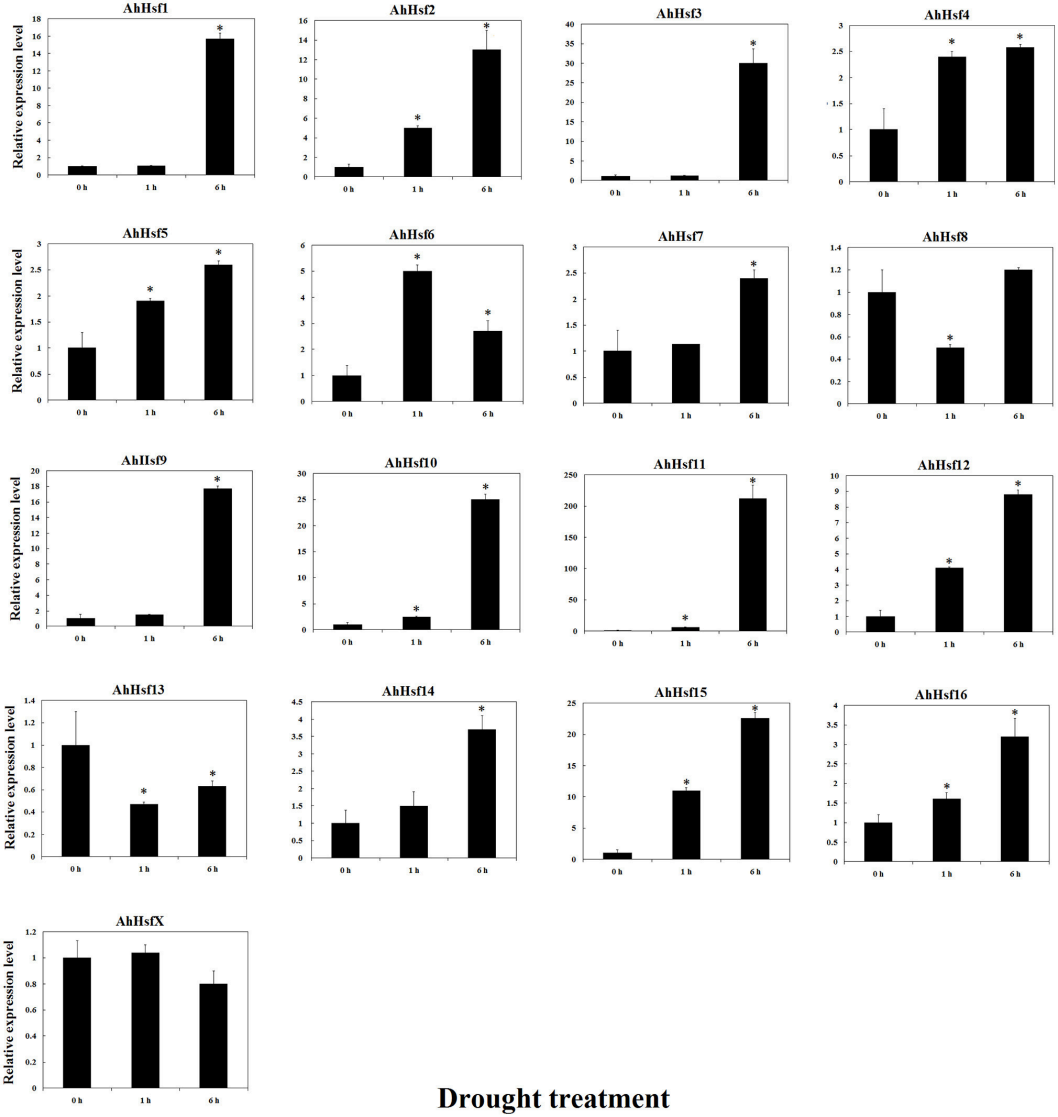
**Relative expression levels of ***Hsfs*** under drought stress in cultivated peanut**. *T*-test was used to perform analysis of significance. ^*^ represents significantly difference (*P* < 0.05) compared with control (0 h).

The expression of most *AhHsfs* was up-regulated under drought stress. The expression levels of *AhHsf2, 4, 5, 7, 12, 14, 15, and 16 were* increased after 1 h of drought treatment. The expression of *AhHsf2, 5, 12, 14, 15*, and *16* was continuously increased during the first 6 h of drought treatment. The expression of *AhHsf1, 3, 9, 10*, and *11* was up-regulated after 6 h of drought stress (~15-folds). *AhHsfX* didn't respond much to drought stress (Figure 8).

The expression of most *AhHsfs* was up-regulated after 1 h of 4°C treatment, and then down-regulated at 6 h after treatment. The expression of *AhHsf 12* was continuously up-regulated during 6 h of cold treatment. The expression of *AhHsf14 was* decreased at 1 h and then increased at 6 h after 4°C treatment (Figure S3).

**Figure 9:**
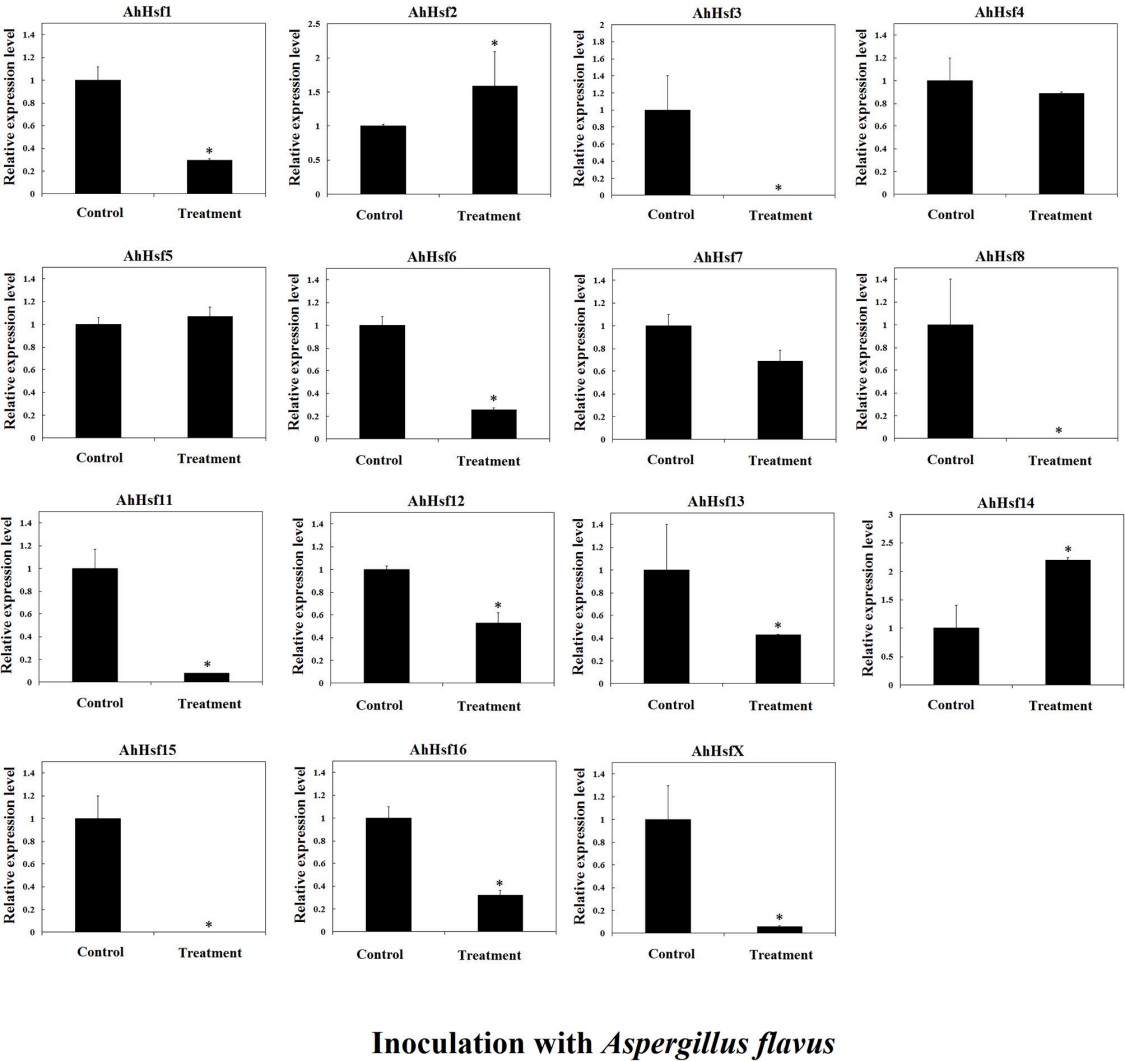
**Relative expression variation of ***Hsfs*** in seeds inoculated with ***Aspergillus flavus*** in cultivated peanut**. *T*-test was used to perform analysis of significance. ^*^ represents significantly difference (*P* < 0.05) compared with control (0 h).

Previous study showed that *Hsfs* may be involved in disease resistance (Pick et al., 2012). In this study, we analyzed the expression of *AhHsfs* in peanut seeds after *A. flavus* infection. The expression of most *AhHsfs* was down-regulated in seed after *A. flavus* inoculation, while the expression of *AhHsf2* and *14* was up-regulated (~1.5-fold; Figure 9).

## Discussions

### Leguminous contained different *Hsf* clusters

B5 cluster was not presented in Arabidopsis Hsfs, while B5 cluster was identified in most leguminous species, such as *C. cajan, L. japonicus* wild peanuts, and *G. max*. B5 Hsf cluster were not detected in *Medicago truncatula*. Phylogenetic tree showed that the leguminous plants contained different Hsf group members. Both in AA and BB wild peanut species, A3, A6a, A7, B3, and B4 cluster members were not found. Only soybean and *M. truncatula* contained the B3 members. A6a and A7 Hsf cluster was not found in leguminous. A3 cluster was not found in wild peanuts and *M. truncatula*. Group C Hsfs were not found in *L. japonicus* and *M. truncatula*. Soybean contained most clusters but not A6a and A7. The number of *Hsfs* from wild peanut species was relative small to compare with cotton, soybean and rosaceae (Li et al., 2014; Wang et al., 2014; Qiao et al., 2015). Phylogenetic tree showed that *A. duran*ensis is the closer relative of *A. ipaensis* compared with other Leguminous.

### WGD may not the major driving force of *Hsfs* large scale expansion in *Arachis*

Our results showed that *Hsf* gene duplication occurred in both AA and BB peanut genomes. The majority pf *Hsf* duplication events were similar between AA and BB genomes. For example, the duplicated gene pair AdHsf7-AdHsf8 was located on chromosome 5. The distance between AdHsf7 and AdHsf8 was about 1 kb. The duplicated gene pair AiHsf7-AiHsf8 was located on chromosome 5, and the distance between these two genes was about 2 kb. However, *AiHsfX* was located on chromosome 6 of BB peanut and its duplicated gene *AiHsf15* was on chromosome 9 of BB peanut. It is possible that *AdHsf15* didn't undergo duplication or the orthologous of *AdHsfX* was lost during the evolution (Figure 3). We only found one tandem duplication gene pairs in *A. duranensis* and *A. ipaensis*, respectively. Both AA and BB genomes or their common ancestor were underwent the early papilionoid whole-genome duplication (WGD) about 58 million year ago (*Ks* = 0.65) (Bertioli et al., 2016). Intraspecies synteny analysis showed that *Hsf* duplication in wild peanut species was not originated from a large scale duplication event, because no intraspecies synteny blocks containing *Hsfs* was found. However, the recent WGD could be a driving force for the expansion of Hsf gene family in Chinese white pear and apple (Qiao et al., 2015). That may be the reason why peanut has less *Hsfs* than that in cotton, soybean and rosaceae (Li et al., 2014; Wang et al., 2014; Qiao et al., 2015).

### *Hsfs* is different in Group B from that in Group A

Group B *Hsfs* underwent positive selection (Table S4). Positive selection could contribute to adaptive evolution, functional diversity, and neofunctionalization (Beisswanger and Stephan, 2008). Study on barley showed that many gene families involved in adaptation to environment were under positive selection. Positive selection may lead to the expansion of these gene families (Zeng et al., 2015). However, group A *Hsfs* underwent purifying selection. Purifying selection may generate genes with conserved functions or pseudogenization (Zhang, 2003). These results indicated that the function of *Arachis* group A Hsfs may be more conserved and the function of group B Hsfs may be more diverged. The sequences of Hsf group B HR-A/B were not conserved compare with group A HR-A/B which was in agreement with the differential selection they experienced (Figure 4). The 3D structure of peanut group B Hsfs was different from group A and C Hsfs. The 3D structure of group A and C HR-A/B was a continuous helix, while group B HR-A/B 3D structure contained helixes which were linked by a linear part (Figure 5).

### The possible roles of *Arachis Hsfs* in abiotic and biotic stresses

Hsfs play a central role in protecting plants from high temperature or other stresses (Nishizawa-Yokoi et al., 2009; Scharf et al., 2012). Many Hsfs could regulate a set of heat-shock protein genes to enhance the thermo-tolerance in plants. Some Hsfs could be regulated by DREB genes as part of drought stress signaling pathway, and enhance drought tolerance (Scharf et al., 2012; Ma et al., 2015; Guo et al., 2016). Some Hsfs could regulate WRKY transcription factors which are involved in response to abiotic stresses, such as drought and cold (Ren et al., 2010; Zou et al., 2010; Jiang et al., 2012; Shen et al., 2015). Arabidopsis *HsfA9* could be activated by ABI3 to enhance seed desiccation tolerance and longevity (Verdier et al., 2013).

In our study, the majority of *Hsf* promoters contained HSE elements (Table S5) suggesting that peanut *Hsfs* could be regulated by other Hsfs. Many peanut *Hsf* promoters contained MYB binding sites which are involved in drought response (Table S5). It indicated that peanut *Hsfs* could be regulated by MYB transcription factors under drought stress. Many *Arachis Hsf* promoters contained ABRE and DRE elements which are involved in ABA-dependent or independent stress tolerance (Chen et al., 2012). Therefore, Hsfs could play important roles for gene regulation in response to different stresses in peanuts. Some *Arachis* Hsf promoters contained salicylic acid responsive, MeJA-responsive or fungal elicitor responsive elements, suggesting their roles in response to pathogen infection.

In cultivated peanut cultivars, the expression level of *AhHsf13* was approximately 500-folds as high as the control after 1 h of heat treatment, and then the expression was decreased after 6 h of treatment. Expression levels of *AhHsf1, 3, 9*, and *AhHsfX* were up-regulated by about 10-folds after 6 h of heat treatment to compare with the control. The expression of these *Hsfs* kept at a high level under continuous heat stress (Figure 7). Group A1a Hsfs were master regulators for acquired thermo tolerance in tomato and Arabidopsis (Scharf et al., 2012). However, we found that the expression of AhHsf2 (group A1) did not respond to heat and cold, but to drought stress. In cultivated peanut, expression levels of *AhHsf1, 2, 3, 9, 10, 11, 15* were about 10-folds as high to compare with the control after 6 h of drought stress (Figure 8). The expression of some *Hsfs* was altered after *Podosphaera aphanis* inoculation in woodland strawberry (Hu et al., 2015). *Aspergillus flavus* produces potent mycotoxin known as aflatoxin which is a key issue of food safety in peanut (Zhang et al., 2015). We detected whether peanut Hsf genes were involved in the response to *A. flavus* infection. The results showed that the expression of *AhHsf2* and *AhHsf14* were significantly up-regulated after *A. flavus* inoculation. The expression of some *AhHsfs* was down-regulated by *A. flavus* infection.

### *Hsf* gene family were highly expressed in peanut seed

Some Hsfs play key roles in plant seed development (Wang et al., 2014). In sunflower and Arabidopsis, *HsfA9* was expressed specifically in seeds and the expression of *Hsp*s was changed during seed development (Almoguera et al., 2002; Kotak et al., 2007b). In rice, *HsfA7* was expressed specifically in seed under normal condition (Chauhan et al., 2011). In peanut, expression levels of more than half of the *AhHsfs* were higher in seeds than that in other tissues. These expression patterns may suggest their roles in peanut seed development.

## Conclusions

Genome-wide identification and comparison of peanut Hsfs with other plant species revealed that peanut contained a small number of *Hsfs*. Phylogenetic tree showed that B5 cluster Hsfs might present only in leguminous. Small scale segmental and tandem duplication but not WGD played important roles in Hsfs expansion in *Arachis*. The sequences of group B Hsf HR-A/B were not conserved compare with group A HR-A/B which was in agreement with the different selection pressure they experienced. We built the 3D structures of peanut Hsfs with the newly submitted templates and found the difference between group A and B members. Peanut *Hsfs* may play important roles in abiotic and biotic stress tolerance based on their expression responses to these stresses.

## Author contributions

XW designed the study, wrote the manuscript and finalized the figures and tables. PW and LH carried out the majority of experiments, data analysis, and wrote the method section of the manuscript. HS, CL, PL, AL, and HG performed experiments.

### Conflict of interest statement

The authors declare that the research was conducted in the absence of any commercial or financial relationships that could be construed as a potential conflict of interest.

## Supplementary material

Figure S1 **Structure of peanut ***Hsfs*****.

Figure S2 **DBD domain in peanut Hsfs**.

Figure S3 **Relative expression levels of ***Hsfs*** under cold stress in cultivated peanut**. *T*-test was used to perform analysis of significance. “*” represents significantly difference (*P* < 0.05) compared with control (0 h).

Table S1 *****Hsfs*** identified in other species**.

Table S2 **Primers of cultivated peanut ***Hsfs*** for qRT-PCR**.

Table S3 **Identity of wild peanut ***Hsf*** orthologous**.

Table S4 **Likelihood values and parameter estimate for wild peanut ***Hsfs*****.

Table S5 **Cis-acting regulatory elements of wild peanut ***Hsf*** promoters**.

Table S6 **Identity of wild and cultivated peanut ***Hsfs*****.
