# Supplementary figures and images for: Genome-Wide Dissection of the Heat Shock Transcription Factor Family Genes in Arachis
Source: Front Plant Sci. 2017 Feb 6;8:106. doi: 10.3389/fpls.2017.00106 (PMC5292572; doi:10.3389/fpls.2017.00106)

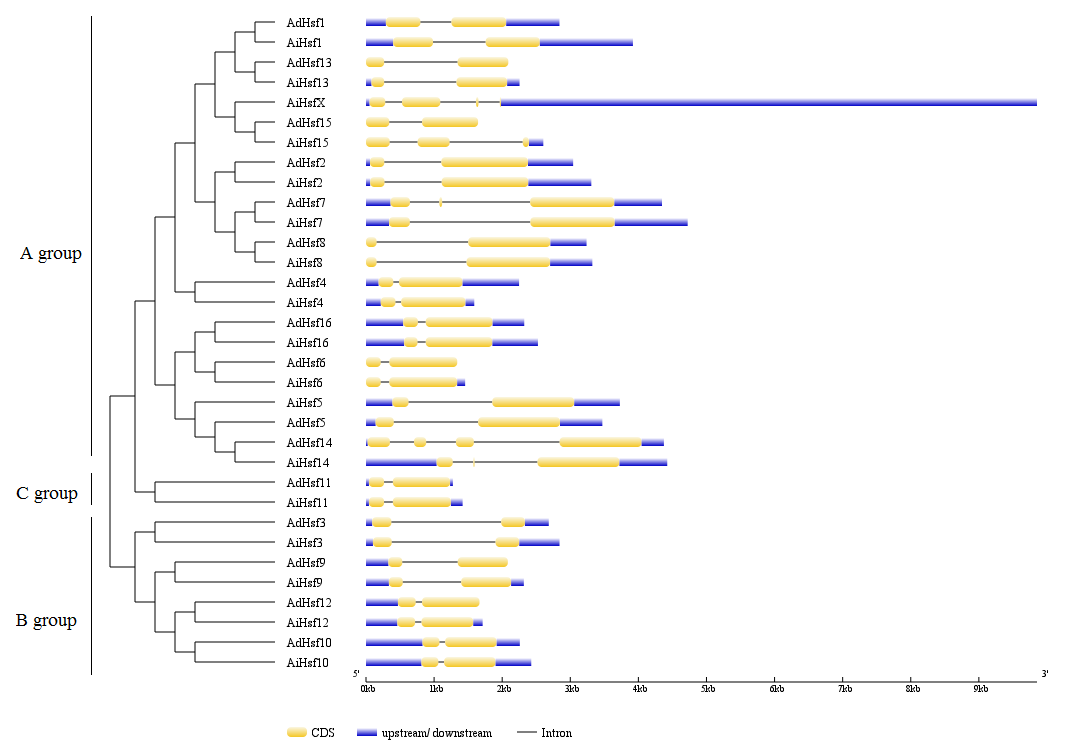

Supplement: Figure S1 — Structure of peanut Hsfs. [file Image1.PNG]

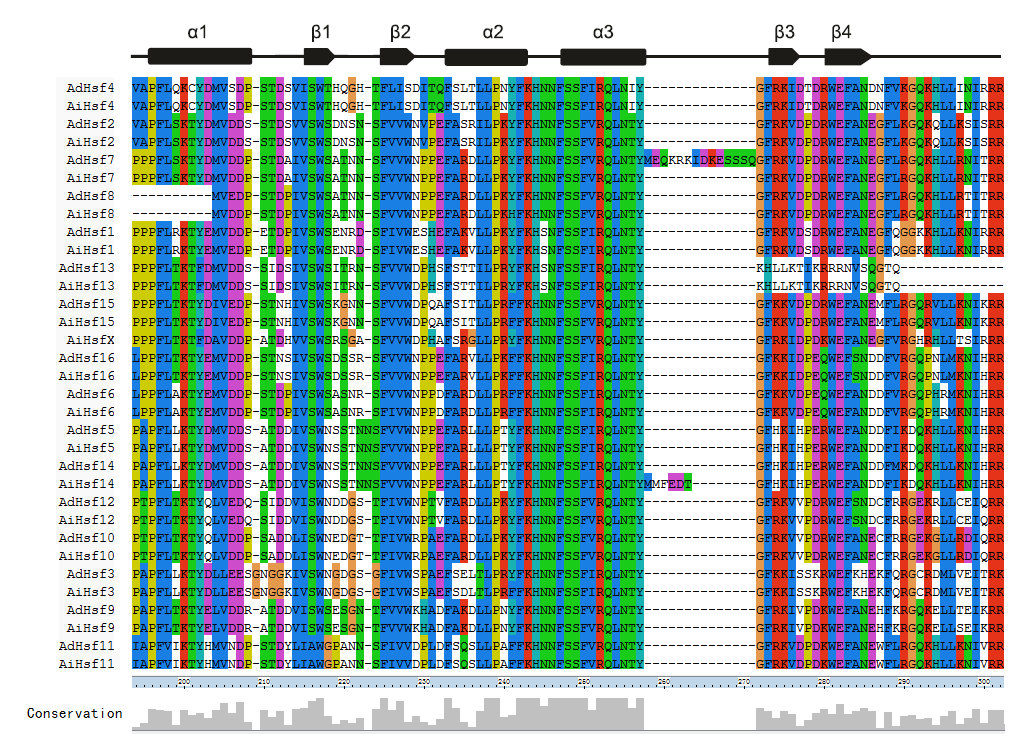

Supplement: Figure S2 — DBD domain in peanut Hsfs. [file Image2.TIF]

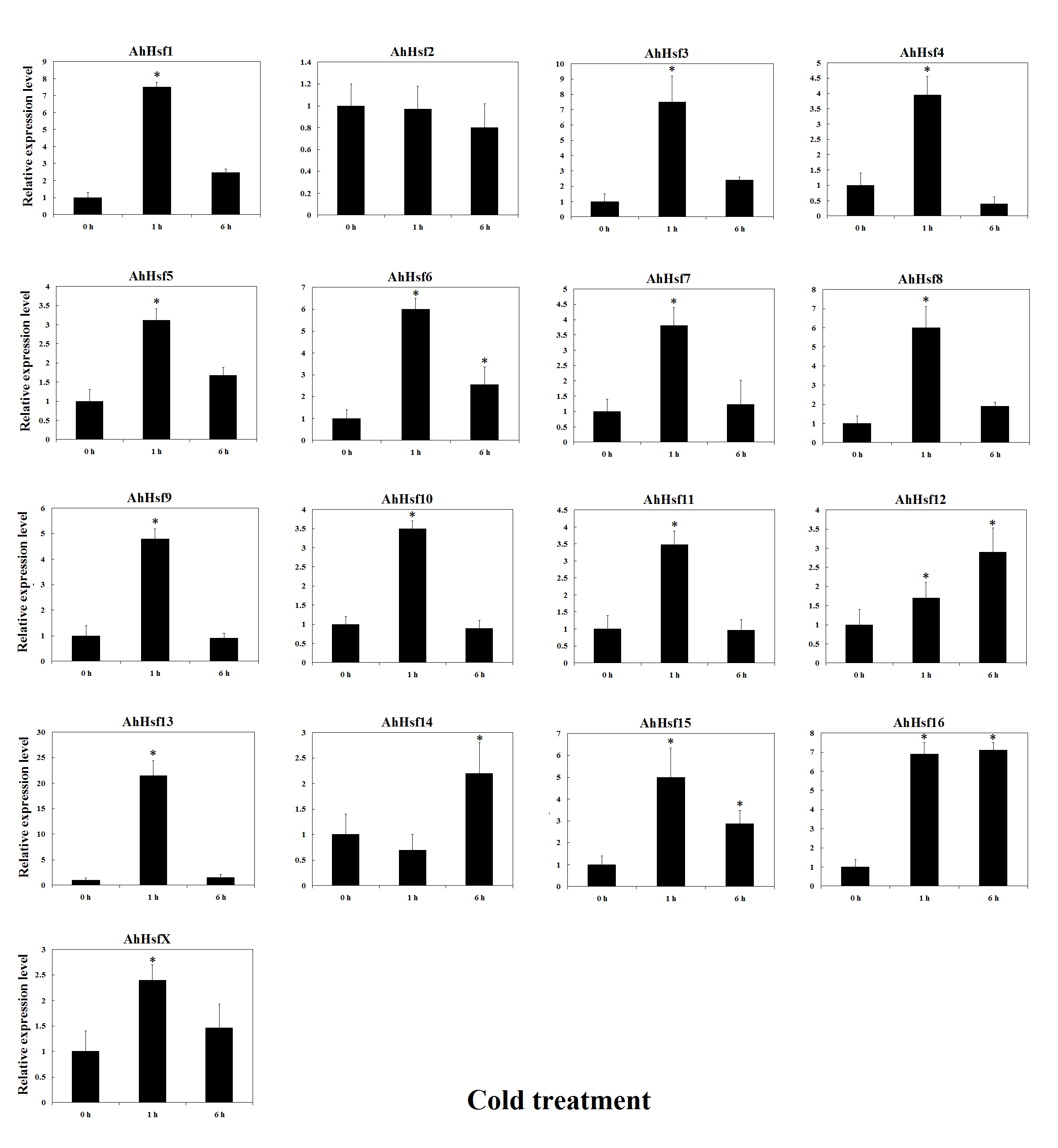

Supplement: Figure S3 — Relative expression levels of Hsfs under cold stress in cultivated peanut. T-test was used to perform analysis of significance. “*” represents significantly difference (P < 0.05) compared with control (0 h). [file Image3.TIF]
